# Differential Cranial Nerve Susceptibility to Compression by a Giant Cerebellopontine Angle Epidermoid Cyst: A Case Report and Literature Review

**DOI:** 10.7759/cureus.107093

**Published:** 2026-04-15

**Authors:** Jesús Oswaldo Díaz Lagunas, Alejandro Becerril-Mejía, Rogelio Revuelta, Gabriel Aco Cruz, Humberto Flores-Velasco, Maria del Cielo Bahena-Quinto, Yael Beristain, Edith Michelle Herrera-Mejía

**Affiliations:** 1 Functional Neurosurgery, National Institute of Neurology and Neurosurgery "Manuel Velasco Suárez", Mexico City, MEX; 2 Neurosurgery, National Institute of Neurology and Neurosurgery "Manuel Velasco Suárez", Mexico City, MEX; 3 Anesthesiology, Mexican Social Security Institute, Pachuca, MEX; 4 Faculty of Medicine, Autonomous University of the State of Morelos, Morelos, MEX; 5 Faculty of Medicine, Instituto Politecnico Nacional, Mexico City, MEX

**Keywords:** cerebellopontine angle epidermoid, giant epidermoid cyst, neurovascular compression, root entry zone, trigeminal neuralgia

## Abstract

Giant cerebellopontine angle (CPA) epidermoid cysts may envelop multiple cranial nerves, yet patients often present with clinically isolated trigeminal neuralgia. The anatomical basis for this selective vulnerability is incompletely appreciated in routine clinical practice.

A 48-year-old man presented with a five-year history of progressive left-sided trigeminal neuralgia involving the maxillary and mandibular divisions. Pain had become severe and refractory to high-dose carbamazepine, with substantial functional and psychological decline. Neurological examination was otherwise unremarkable, with intact facial nerve function and normal hearing on preoperative audiometry.

Brain magnetic resonance imaging revealed a 6.1 cm left CPA epidermoid cyst circumferentially involving the trigeminal, facial, and vestibulocochlear nerves, without extension into the internal auditory canal. Despite this extensive radiological involvement, clinical dysfunction was confined to the trigeminal nerve. The patient underwent retrosigmoid craniectomy with maximal safe resection, prioritizing decompression of the trigeminal root entry zone while leaving capsular remnants adherent to cranial nerves VII and VIII to preserve neural function.

Trigeminal pain resolved completely and immediately after surgery, allowing discontinuation of all analgesic medication. A mild high-frequency sensorineural hearing loss was the only postoperative deficit, with preservation of functional hearing.

The selective trigeminal vulnerability observed in this case reflects the proximal location of the trigeminal central-peripheral myelin transition zone, which overlaps the root entry zone and is therefore exposed earliest to compressive forces from expanding CPA lesions. This case supports individualized surgical strategies that prioritize symptomatic decompression over radical resection in giant CPA epidermoid cysts.

## Introduction

Epidermoid cysts are histologically benign, slow-growing, extra-axial lesions that arise from ectodermal cell inclusions misplaced during early embryogenesis. They account for approximately 0.2-1.8% of all intracranial tumors, with a marked predilection for the cerebellopontine angle (CPA), which harbors 40-50% of cases [1]. These lesions enlarge by progressive desquamation and accumulation of keratinaceous material within the cyst cavity, typically following subarachnoid spaces and enveloping neurovascular structures rather than displacing them. As a result, they can reach large sizes before producing symptoms, which usually arise from cranial nerve or brainstem compression rather than parenchymal invasion [2].

According to the third edition of the International Classification of Headache Disorders (ICHD-3) [3], trigeminal neuralgia (TN) is defined as recurrent, unilateral, brief episodes of electric shock-like or lancinating facial pain confined to one or more divisions of the trigeminal nerve, precipitated by innocuous stimuli and lasting less than two minutes [4]. Secondary TN is attributed to an underlying structural lesion, such as a tumor or demyelinating plaque, and tends to occur at a younger age than classical neurovascular compression syndromes. In the CPA, epidermoid cysts are a well-recognized cause of secondary TN, often in the absence of other overt cranial nerve deficits [1].

Giant intracranial epidermoid cysts, generally defined as lesions exceeding 5 cm in maximum diameter, are uncommon but clinically important because they may involve multiple cranial nerves, the brainstem, and major arterial trunks [5]. Interestingly, even when several cranial nerves are radiologically compressed or encased, the clinical presentation may be dominated by a single hyperactive dysfunction syndrome, such as TN, whereas other cranial nerve deficits are mild, subclinical, or absent [6]. This observation raises a key mechanistic question: why is the trigeminal nerve preferentially susceptible to compressive pathology compared with other cranial nerves sharing the same confined anatomical space?

The present report describes a patient with medically refractory secondary TN caused by a giant CPA epidermoid cyst that circumferentially involved all ipsilateral cranial nerves but produced clinically isolated trigeminal dysfunction. Building on this case, a focused review of the literature is used to analyze cranial nerve anatomy, particularly the central-peripheral myelin transition zone, and to elucidate structural and pathophysiological factors that may underlie the differential susceptibility of CPA cranial nerves to compressive lesions.

## Case presentation

A 48-year-old right-handed man with no significant past medical or surgical history and no family history of neurological disease was referred to our institution for evaluation of medically refractory left-sided facial pain.

The patient reported that his symptoms began approximately five years before presentation and were characterized by paroxysmal unilateral facial pain localized to the maxillary (V2) and mandibular (V3) divisions of the trigeminal nerve (CN V). Episodes were described as brief, electric shock-like attacks lasting less than two minutes and occurring more than four times daily. Pain was triggered by light tactile stimulation of the left cheek, toothbrushing, chewing, or exposure to cold air, although spontaneous attacks were also reported. Between paroxysms, the patient remained pain-free. Pain severity was rated 7/10 on the Numerical Rating Scale (NRS) [7] and classified as Barrow Neurological Institute (BNI) [8] pain intensity score III (some pain, adequately controlled with medication). The clinical presentation fulfilled the ICHD-3 diagnostic criteria for TN. Initial pharmacological management with carbamazepine 400 mg/day achieved adequate pain control without significant adverse effects.

Over the following five years, the patient experienced progressive worsening of symptoms despite escalation of pharmacological therapy. By the time of surgical evaluation in 2025, paroxysmal frequency had increased to more than eight attacks per day, and pain intensity had reached NRS 10/10. Each paroxysm was followed by lower-intensity continuous aching pain lasting up to two hours, consistent with TN with concomitant continuous pain as defined by the ICHD-3 criteria. Despite escalation of carbamazepine to 1200 mg/day, pain was no longer controlled, and adverse effects, including persistent dizziness and daytime somnolence, developed. Pain severity was reclassified as BNI V (severe pain or no pain relief). The patient reported avoidance of eating, speaking, and social interactions due to fear of triggering attacks and subsequently developed clinically significant anxiety and depressive symptoms. Functional status declined substantially, with a Karnofsky Performance Status (KPS) [9] score of 60, indicating that the patient required occasional assistance and was unable to carry out normal daily activities.

Neurological examination revealed no focal deficits. Sensation to light touch and pinprick was intact across all three divisions of the CN V. The corneal reflex was present bilaterally, and masseter muscle strength was normal. Facial nerve function was preserved and classified as House-Brackmann grade I (normal function). Examination of the remaining cranial nerves was unremarkable, and cerebellar testing showed no abnormalities. No long-tract signs were identified.

**Figure 1:**
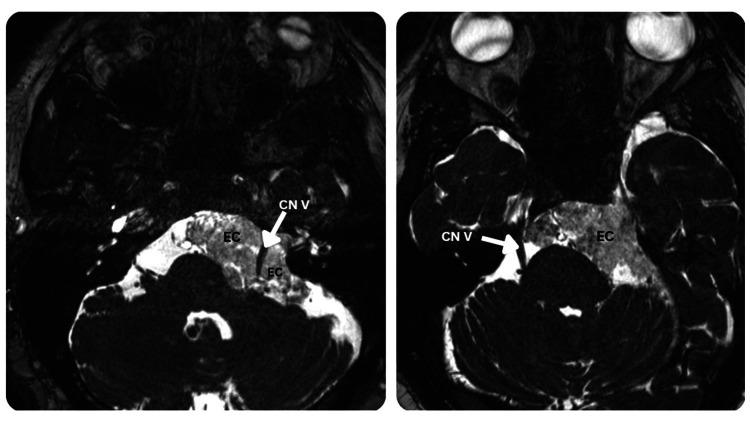
Axial T2-weighted MRI showing a cerebellopontine angle epidermoid cyst causing trigeminal nerve compression Axial T2-weighted images show a hyperintense lesion in the cerebellopontine cistern consistent with an EC. The lesion extends along the cerebellopontine angle and compresses the CN V at its root entry zone along the lateral surface of the pons. White arrows indicate the trigeminal nerve displaced by the epidermoid lesion. CN V, trigeminal nerve; EC, epidermoid cyst.

Magnetic resonance imaging (MRI) of the brain with and without gadolinium contrast was obtained, including axial T1-weighted, T2-weighted, fluid-attenuated inversion recovery (FLAIR), diffusion-weighted imaging (DWI), and three-dimensional fast imaging employing steady-state acquisition sequences. Imaging revealed a large lobulated extra-axial lesion in the left CPA measuring approximately 6.1 cm in maximal diameter. The lesion followed cerebrospinal fluid signal intensity on T1- and T2-weighted sequences, demonstrated heterogeneous signal on FLAIR, and showed marked hyperintensity on DWI, consistent with an epidermoid cyst. No contrast enhancement was observed. The lesion displayed characteristic irregular margins conforming to the subarachnoid spaces without extension into the internal auditory canal (Figure 1). The mass displaced the trigeminal nerve superiorly and displaced cranial nerves VII and VIII posteroinferiorly, producing mild compression of the lateral pons and the left cerebellar hemisphere. No obstructive hydrocephalus or additional intracranial lesions were identified.

As part of the preoperative evaluation, the otoneurology service performed comprehensive audiological testing. Pure-tone audiometry demonstrated normal hearing bilaterally, with air-conduction thresholds between 0 and 25 dB hearing level across frequencies from 250 to 8000 Hz. Speech reception thresholds were consistent with pure-tone averages, and word recognition scores were at least 90% in both ears. Tympanometry demonstrated type A curves bilaterally, confirming normal middle ear function.

**Figure 2:**
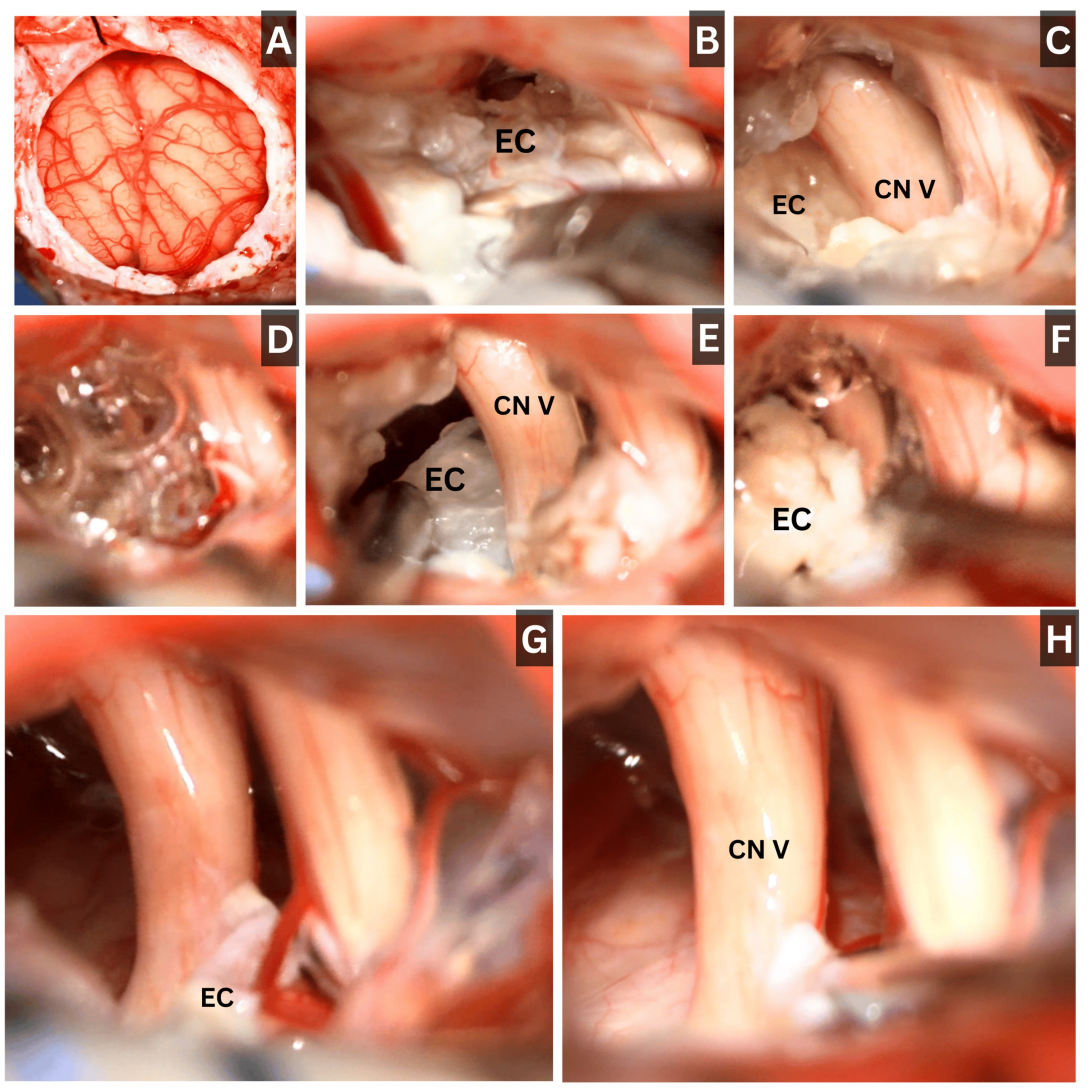
Intraoperative imaging of giant left cerebellopontine angle epidermoid cyst with trigeminal root entry zone compression A) C-shaped dural opening with cerebrospinal fluid drainage, B) arachnoid dissection and exposure of the epidermoid cyst compressing the cranial nerves of the cerebellopontine angle, C) cyst compression of the trigeminal nerve and vestibulocochlear complex, D) hydrodissection for cyst mobilization and rupture, E) suction-assisted cyst debulking, F) combined hydrodissection and suction, G) identification of the trigeminal root entry zone with residual cyst compression, and H) microsurgical cyst dissection achieving trigeminal decompression. CN V, trigeminal nerve; EC, epidermoid cyst.

Given the presence of medically refractory TN (BNI V), imaging findings demonstrating a giant CPA epidermoid cyst producing neurovascular compression and significant functional decline (KPS 60), surgical treatment was indicated. The patient underwent retrosigmoid craniectomy with partial resection of the CPA epidermoid cyst (Figure 2). Intraoperatively, a large pearly white lobulated epidermoid tumor was identified with dense capsular adhesions to the trigeminal nerve at the root entry zone (REZ), as well as to cranial nerves VII and VIII, the anterior inferior cerebellar artery, and the superior cerebellar artery. Maximal safe resection was achieved using meticulous microsurgical dissection, resulting in complete decompression of the trigeminal REZ. Capsular remnants adherent to cranial nerves VII and VIII were intentionally left in situ to minimize the risk of iatrogenic cranial neuropathy. Copious irrigation with warm saline was performed to reduce the risk of chemical meningitis caused by keratin debris spillage.

The patient reported complete and immediate resolution of TN upon awakening from anesthesia. Postoperative pain was classified as BNI I (no trigeminal pain, no medication) with an NRS score of 0/10, and all analgesic and anticonvulsant medications, including carbamazepine, were discontinued on the first postoperative day. Early postoperative neurological examination demonstrated no new deficits and preserved facial nerve function (House-Brackmann grade I). Postoperative audiological assessment revealed new mild left-sided sensorineural hearing loss, characterized by slightly elevated thresholds at higher frequencies (4000-8000 Hz). Speech reception thresholds remained consistent with pure-tone averages, and word recognition scores were at least 90% bilaterally, indicating preservation of functional hearing. This deficit was attributed to the manipulation of cranial nerve VIII during the dissection of adherent capsular fragments.

At the most recent follow-up, the patient remained completely pain-free (BNI I, NRS 0/10) without the need for medication. Functional status improved substantially, with the KPS increasing from 60 preoperatively to 90 postoperatively, reflecting a return to near-normal daily activity. The patient also reported marked improvement in psychological well-being and a subjective return to his premorbid quality of life.

## Discussion

Differential cranial nerve susceptibility: the central question

The most striking feature of this case is the discordance between the radiological burden of disease and the clinical phenotype. Despite circumferential involvement of all ipsilateral CPA cranial nerves by a 6.1-cm epidermoid cyst, the patient presented exclusively with TN, while cranial nerves VII, VIII, and IX remained clinically intact. This pattern is not unique to the present case; a series of CPA epidermoid cysts reported that dysfunction occurred most often in cranial nerve VIII (73%), cranial nerve V (56%), and cranial nerve VII (24%), yet the majority of patients presented with hyperactive dysfunction, principally TN, rather than pure loss-of-function deficits [10,11]. Understanding why the trigeminal nerve is preferentially vulnerable requires examination of the microstructural, anatomical, and pathophysiological factors that converge at the REZ.​

The transition zone is the site of vulnerability

Every cranial nerve contains a short segment where central myelin produced by oligodendrocytes gives way to peripheral myelin produced by Schwann cells. This central-peripheral transition zone is structurally discontinuous and has limited capacity for repair, which makes it the most mechanically vulnerable part of the nerve. The location and length of this transition zone differ markedly between cranial nerves, and these differences correlate with the incidence of the corresponding neurovascular compression syndromes [12].

Comparative anatomy of the TZ across CPA cranial nerves provides a compelling framework for understanding differential susceptibility (Table 1).

**Table 1:** Comparative anatomy of the central-peripheral myelin TZ and epidemiology of NVCS across cerebellopontine angle cranial nerves IAC, internal auditory canal; NVCS, neurovascular compression syndrome; REZ, root entry zone; TZ, transition zone.

Cranial nerve	Predominant function	Specimens (n)	TZ distance from brainstem, mean ± SD (mm)	TZ location relative to REZ	Cisternal length, mean (range) (mm)	Associated NVCS	NVCS annual incidence (/100,000)	Reference
CN V	Sensory (80%)	100 nerves	Medial: 1.13 ± 0.45; lateral: 2.47 ± 1.23	Proximal; overlaps REZ	12.3 (8-15)	Trigeminal neuralgia	4-29	[13]
CN VII	Motor (90%)	75 nerves​; 12 cadavers	Medial: 0.58; lateral: 1.9; medial REZ length: 2.6 (1.6-3.5)	Proximal; overlaps REZ	14.8-20.9	Hemifacial spasm	1.53	[14]
CN VIII	Sensory (100%)	30 nerves	11.50 ± 1.56 (9.28-13.84)	Distal; does NOT overlap REZ; near IAC	14.2-19.2 ​	Vestibular paroxysmia	<50 (rare; <1/2000 prevalence)	[15]
CN IX	Mixed	12 nerves	1.51 ± 0.39	Proximal; overlaps REZ (cone shaped)	14.2-19.9 ​	Glossopharyngeal neuralgia	0.7	[16,17]

Several observations arise from this comparison. First, the TZ of the trigeminal nerve is located proximally, within 1.13 to 4.19 mm of the brainstem, and overlaps the REZ, making it the first structure subjected to compressive forces from an expanding CPA mass. Second, regional variability in the extent of central myelination within the trigeminal nerve itself (shorter medially than laterally) may further influence local susceptibility, as the medial portion encounters vascular and tumoral compression earlier. Third, the TZ of the vestibulocochlear nerve is located distally (9.28-13.84 mm from the brainstem), near the internal auditory canal, and does not overlap the REZ. This distal position explains why CPA epidermoid cysts, which characteristically conform to subarachnoid spaces without extending into the internal auditory canal, may compress the proximal cisternal segment of cranial nerve VIII without reaching its TZ [14,18].​

In the present case, the epidermoid cyst displaced cranial nerves VII and VIII posteroinferiorly but did not extend into the internal auditory canal, consistent with the typical growth pattern of these lesions. Thus, although compression was present, it occurred in the peripheral myelin segment of the vestibulocochlear nerve, sparing the distal TZ. By contrast, the trigeminal nerve was displaced superiorly with direct compression at its proximal TZ, the precise location where compressive pathology is most likely to produce symptomatic dysfunction.

Pathophysiology of compression-induced TN

The dominant pathophysiological model posits that sustained compression at the TZ initiates a cascade of focal demyelination, ectopic impulse generation, and ephaptic crosstalk between neighboring fibers. Demyelination of primary sensory trigeminal afferents at the REZ disrupts axoglial junctions and sodium channel clustering, creating zones of hyperexcitability that serve as ectopic pacemakers [15]. Tactile signals carried by fast myelinated A-beta fibers can then aberrantly activate slow nociceptive A-delta fibers through ephaptic transmission, giving rise to the characteristic high-frequency paroxysms of electric shock-like pain [13,15].

Voltage-gated sodium channels are central to this process. Changes in Nav1.3 and Nav1.7 expression have been documented in compressed trigeminal afferents, and these molecular changes lower the threshold for action potential generation and sustain ectopic firing [19]. The clinical efficacy of carbamazepine and oxcarbazepine, which stabilize sodium channels in their inactivated state, indirectly supports this mechanism [15]. In the present case, failure of high-dose carbamazepine likely reflected mechanical deformation that exceeded what pharmacological modulation alone could compensate for.

Immediate and complete resolution of pain following microsurgical decompression of the trigeminal REZ in this patient establishes a direct causal relationship between structural compression and electrophysiological instability, consistent with prior series in which 87 to 98% of patients experience prompt pain relief after microvascular decompression [16].​

Giant epidermoid cysts: surgical considerations and literature review

Giant intracranial epidermoid cysts pose unique surgical challenges. The retrosigmoid approach remains the preferred corridor for CPA epidermoid cysts, providing direct access to the trigeminal REZ and facilitating cyst decompression [11,17]. Maximal safe resection is considered the standard of care; however, dense capsular adhesions to cranial nerves and vascular structures, as observed in the present case, often preclude complete excision. In a series of 28 skull base epidermoid cysts, 64% underwent gross total resection, but the remaining 36% required subtotal resection due to adherence to critical structures; importantly, no patients experienced permanent worsening of cranial nerve function. Residual capsular remnants are associated with recurrence at a mean interval of 3.6 to 7.7 years, supporting the need for indefinite MRI surveillance [20]. Capsule strangulation of cranial nerves represents a distinct mechanism of nerve dysfunction, particularly in younger patients with rapidly progressive deficits [17].​

Spillage of keratinous cyst contents into the subarachnoid space during resection may provoke aseptic chemical meningitis, reported in 2 to 50% of cases [21]. In the present case, copious irrigation with warm saline was performed to mitigate this risk. Malignant transformation of epidermoid cysts to squamous cell carcinoma, although exceedingly rare, has been documented and provides an additional argument for maximal safe resection when feasible [10,22].​

The postoperative complication of mild sensorineural hearing loss in this case, despite preserved preoperative audiometry, warrants specific discussion. Intraoperative manipulation of capsular remnants adherent to cranial nerves VII and VIII carries an inherent risk of cochlear nerve injury. The decision to leave adherent capsular fragments on these nerves reflects a deliberate strategy to prioritize neural preservation over radical excision. Postoperative word recognition scores remained at or above 90% bilaterally, indicating functional hearing preservation despite mild threshold elevation at higher frequencies.​

Why not hemifacial spasm, vestibular paroxysmia, or glossopharyngeal neuralgia?

The absence of hemifacial spasm in this case is notable, given the documented compression of cranial nerve VII. Hemifacial spasm results from compression of the facial nerve at its proximal TZ, located 1.9 to 2.86 mm from the brainstem, and overlapping the REZ. The facial nerve is predominantly motor, and its compression syndrome manifests as involuntary contractions of facial musculature rather than pain. In the present case, House-Brackmann grade I [23] function was documented pre- and postoperatively, suggesting that the compressive force exerted by the epidermoid cyst was insufficient to produce focal demyelination at the facial nerve TZ, or that the vector of displacement (posteroinferior) did not concentrate mechanical stress at the proximal TZ.

The absence of vestibular paroxysmia or cochlear symptoms preoperatively is explained by the distal location of the vestibulocochlear nerve TZ (9.28-13.84 mm from the brainstem), which does not overlap the REZ and is situated near the internal auditory canal [16]. Because the epidermoid cyst conformed to the subarachnoid spaces without entering the internal auditory canal, compression occurred exclusively in the proximal cisternal segment composed of peripheral Schwann cell myelin, which is inherently more resistant to compressive injury than central myelin. This anatomical arrangement favors preservation of vestibular and cochlear function until much later stages of disease.

The glossopharyngeal nerve has the shortest TZ among CPA cranial nerves (approximately 1.51 mm), yet glossopharyngeal neuralgia has the lowest incidence (0.2-0.7 per 100,000) [16]. This apparent paradox is likely explained by the small volume of central myelin and the relatively limited arterial and tumoral contact area within the vulnerable segment. In the present case, the absence of glossopharyngeal neuralgia is consistent with the low baseline probability of symptomatic compression at this nerve, even in the context of a giant CPA mass.​

Limitations

This report has several limitations inherent to the case report design. The findings are derived from a single patient and cannot be generalized to all patients with CPA epidermoid cysts. The review of cranial nerve susceptibility relies on anatomical data obtained from cadaveric specimens, which may not perfectly reflect in vivo measurements due to postfixation tissue shrinkage estimated at up to 20% [14]. Intraoperative neurophysiological monitoring data, which could have provided direct electrophysiological evidence of trigeminal demyelination and real-time assessment of cranial nerve integrity, were not available for analysis. Additionally, follow-up is limited, and long-term outcomes regarding cyst recurrence and the evolution of the sensorineural hearing loss remain to be determined.​

## Conclusions

This case demonstrates that a giant CPA epidermoid cyst can circumferentially involve all ipsilateral cranial nerves yet produce clinically isolated TN. The preferential susceptibility of the trigeminal nerve reflects the proximal location of its central-peripheral myelin transition zone, its overlap with the REZ, and the downstream consequences of focal demyelination and sodium channel dysregulation under sustained compressive stress. Comparative anatomical analysis of transition zone length and position across CPA cranial nerves provides a structural basis for the differential clinical expression of compressive pathology. Microsurgical decompression of the trigeminal REZ achieved immediate and complete pain relief in this patient, supporting a direct causal link between mechanical deformation and electrophysiological instability. Giant epidermoid cysts of the CPA thus serve as a macroscopic model of cranial nerve REZ dysfunction and highlight the importance of individualized surgical strategies that prioritize maximal safe decompression while preserving adjacent neural structures.
